# Barriers and enablers to managing challenging behaviours after traumatic brain injury in the acute hospital setting: a qualitative study

**DOI:** 10.1186/s12913-023-10279-z

**Published:** 2023-11-16

**Authors:** Heather Block, Michelle Bellon, Sarah C. Hunter, Stacey George

**Affiliations:** 1 Caring Futures Institute, College of Nursing and Health Science, Flinders University, Adelaide, Australia; 2 Division of Allied Health, Flinders Medical Centre, Southern Adelaide Local Health Network, Adelaide, Australia

**Keywords:** Traumatic brain injury, Challenging behaviour, Implementation, Staff, Qualitative research, Acute care

## Abstract

**Background:**

Challenging behaviours after traumatic brain injury (TBI) in the acute setting are associated with risk of harm to the patient and staff, delays in commencing rehabilitation and increased length of hospital stay. Few guidelines exist to inform practice in acute settings, and specialist services providing multi-disciplinary expertise for TBI behaviour management are predominantly based in subacute inpatient services. This study aims to investigate acute and subacute staff perspectives of barriers and enablers to effectively managing challenging behaviours after TBI in acute hospital settings.

**Methods:**

Qualitative focus groups were conducted with 28 staff (17 from acute setting, 11 from subacute setting) across two sites who had experience working with patients with TBI. Data were analysed using inductive-deductive reflexive thematic analysis. Data were applied to the constructs of the integrated-Promoting Action on Research Implementation in Health Services (i-PARIHS) framework to generate themes representing barriers and enablers to managing challenging behaviours after TBI in the acute hospital setting.

**Results:**

Four barriers and three enablers were identified. Barriers include (1) Difficulties with clinical decision making; (2) Concerns for risks to staff and patients; (3) Hospital environment; (4) Intensive resources are required. Enablers were (1) Experienced staff with practical skills; (2) Incorporating person-centred care; and (3) Supportive teams.

**Conclusion:**

These findings can inform pre-implementation planning for future improvements to TBI behaviour management in acute hospital settings. Difficulties with clinical decision making, concerns for risks of injury, the hospital environment and lack of resources are major challenges. Implementation strategies developed to address barriers will need to be trialled, with multi-disciplinary team approaches, and tailored to the acute setting.

**Supplementary Information:**

The online version contains supplementary material available at 10.1186/s12913-023-10279-z.

## Background

Traumatic brain injury (TBI) has a high incidence internationally, with 69 million individuals estimated to sustain a TBI annually [1]. Motor vehicle accidents are the most common cause of TBI, followed by falls, violence, and injuries from sporting activities [2]. People with TBI can experience a range of physical, sensory, communication, cognitive, behavioural, and psychosocial difficulties [2–5]. Challenging behaviours following TBI can include a range of behavioural disturbances including agitation, irritability, aggression, inappropriate sexual behaviour, perseveration, wandering/absconding, and apathy [6]. Previous studies have estimated challenging behaviours are prevalent in 70–86% of hospitalised TBI patients following their injury [7, 8]. Challenging behaviours after TBI are associated with risk of harm to the patient and staff, delays in commencing rehabilitation and increased length of hospital stay [7, 9–11].

### Effective TBI behaviour management in acute settings

The evidence for the management of challenging behaviours during the acute phase of TBI recovery is equivocal, requiring more research to provide evidence-based treatment recommendations to improve care [12–14]. In the absence of high-quality evidence for the efficacy of TBI behaviour interventions, clinicians are guided by clinical practice guidelines and expert opinion to guide clinical decisions for TBI behaviour management in acute settings [15–18]. Clinical practice guidelines for the management of challenging behaviours in TBI in the acute setting exist, but few guidelines provide comprehensive detail on the implementation of recommendations into clinical care, thereby limiting adoption of evidence into practice [19].

Evidence and guideline recommendations for the management of challenging behaviours after TBI in the acute setting entails: assessment and regular monitoring of behaviour change; non-pharmacological interventions; followed by pharmacological treatments if required [15–17, 19, 20]. TBI Behaviour assessment should include comprehensive, individualised assessment through diagnostic interviews and direct objective observations [15, 21, 22], with identification of differential causes of agitation, for example pain, sepsis, withdrawal and anxiety [16, 18].

Non-pharmacological treatments include environmental modifications (for example minimising stimulation, and a safe and secured environment); behaviour modification techniques (for example consistent staffing, structured care, positive reinforcement strategies); reduced use of restraints; reorientation and cognitive strategies, restoration of sleep-wake patterns; supervised wandering; family involvement, and education for staff and families [14, 15, 20, 23–25]. National policies and standards recommend minimising restrictive practices, as such reducing use of physical and mechanical restraints, for patients in hospital settings [26].

Pharmacological treatment involves treatment with a pharmaceutical component [12], such as antipsychotics/neuroleptics, anti-depressants, psychostimulants, anti-parkinsonians and anti-convulsants [7, 16]. Although high-quality studies to support use of pharmacological interventions for challenging behaviours after acute TBI are lacking [13], guideline recommendations for pharmacological treatment with highest supporting evidence include beta-blockers [15, 16, 18, 19]. Careful drug selection and monitoring when initiating pharmacological interventions to minimise potentially adverse effects is required [15].

Previous studies have identified clinicians use a range of interventions for managing challenging behaviours, particularly agitation after TBI; however, many lack the sufficient training, resources, guidelines and support to feel confident and satisfied in managing agitation [27]. Furthermore, staff working with patients with TBI must anticipate, de-escalate, and cope effectively with aggressive and agitated behaviours while minimising outbursts [20]. Lack of rigorous research on effective TBI behaviour management in the acute setting, insufficient training for clinicians and lack of resources in the acute setting highlights the challenges in delivering consistent and effective care to people with TBI. Therefore, there is a need to explore staff perspectives of managing challenging behaviours after TBI in the acute setting to investigate factors influencing the delivery of effective and quality care.

### Implementation of complex interventions in the acute context

Implementation science recognises that strong evidence alone is not sufficient to change practice in healthcare settings [28]. Translation of evidence, particularly complex or multi-component innovations, into practice can be difficult due to a range of factors that influence the implementation, adaptation, integration, diffusion and sustainability of evidence-based healthcare [29]. Implementation frameworks help us robustly understand the multiple factors to effectively plan and evaluate implementation of evidence into practice within complex environments, systems, and teams. Complex interventions in healthcare are multi-component; target a range of behaviours; require expertise and skills from those delivering the intervention; and flexibility in tailoring to the targeted individual or healthcare setting [30]. Management of challenging behaviours is complex, requiring multi-disciplinary team approaches (commonly including medical, nursing and allied health professionals) with skills, experience and flexibility to identify, adapt and treat a range of behaviour changes within the hospital context [7, 31]. Acute hospitals present unique barriers to implementation including variable patient presentations, complex health services, health conditions, the clinical environment, hospital processes and microsystems [32]. Implementation of quality and safety innovations in hospital settings can be influenced by contextual factors such as clinicians lack of knowledge, time and resources to successfully contribute to improvements in clinical practice [33]. All are complex factors which can influence the effective delivery of TBI behaviour management in the acute setting. Staff perspectives gained through qualitative studies provide valuable insights into the experiences of clinicians within health services, thus informing service development and adaptions for improvements to clinical care in healthcare settings [34]. Within specialised subacute brain injury rehabilitation settings, staff have specialised skills in the physical, cognitive, behavioural and emotional needs of patients recovering from TBI [20] with adequate resources and environment for optimal TBI recovery. In contrast, patients in the early recovery stage of TBI are often cared for in acute neurosurgical or trauma units in hospitals with transient staffing, varied experience, knowledge, and resources relevant to TBI recovery. The perspectives of staff from both acute care and specialised subacute TBI rehabilitation are necessary to elucidate the implementation contextual factors for TBI behaviour management for patients who will often commence their recovery in acute care, then transition to rehabilitation.

Perspectives on challenging behaviours have been described by staff across acute care for dementia [35]; disability [36]; mental health services [37]; emergency departments [38]; and general hospital wards [39]. However, there are few qualitative studies identifying staff perspectives of managing challenging behaviours after TBI in acute hospital settings. Although Oyesanya et al. [40] describe nurses concerns and barriers to caring for acute TBI patients, few studies identify multi-disciplinary staff perspectives specifically relating to acute TBI challenging behaviour management. Carrier et al. [31] interviewed 33 clinicians from 16 countries working with agitated patients in the early recovery of TBI and found effective agitation management during acute TBI continues to pose a significant challenge to clinicians worldwide. Themes highlighted the broad approaches to effective agitation management involved: managing safety; managing triggers of agitation; managing behaviour; clinician influences; and systemic influences [31]. A previous study conducted by our research team involved implementation of a consistent TBI behaviour assessment and management approach across two acute hospital settings [25]. Results found a high level of clinician adherence to the behaviour management approach, with lowered use of restraints and admission costs for the patients with TBI who received the implemented approach [25]. However, there is a lack of research that has systematically applied implementation frameworks to robustly investigate barriers and enablers to managing challenging behaviours after TBI in the acute setting.

### Objective of this study

To elucidate implementation contextual factors, this study focuses on staff’s perspectives of the barriers and enablers of managing challenging behaviours after TBI in the acute setting. The findings can provide pre-implementation planning for future opportunities to inform implementation strategies to address barriers and leverage enablers. To understand the contextual factors within the acute setting to guide future implementation strategies, an implementation framework is necessary. The integrated – Promoting Action on Research Implementation in Healthcare Settings (i-PARIHS) implementation framework focuses on the quality/type of innovation, the individuals or teams, and the characteristics of the context, and how these factors are supported by facilitation [41]. As a hybrid process and determinant implementation framework, i-PARIHS can be used to specify the steps in the process of translating research into practice; and the types of determinants which act as barriers and enablers that influence implementation outcomes [42]. I-PARIHS was selected as the most suitable implementation framework for this study to understand the multiple, complex and intertwined factors relating to barriers and enablers to effectively managing challenging behaviours after TBI in the acute setting.

This current study addresses this gap in the literature with a qualitative investigation of staff perspectives of TBI behaviour management in the acute setting. The aims of this study were to:

1. Investigate acute and subacute staff perspectives of barriers and enablers to managing challenging behaviours after TBI in acute hospital settings.
2. Apply findings to the constructs of the i-PARIHS framework to understand contextual factors to TBI behaviour management in the acute setting.

## Methods

### Design

A qualitative study using focus groups at a major trauma hospital and a subacute specialised inpatient brain injury rehabilitation unit in Australia was conducted. ethics approval was gained through the Southern Adelaide Clinical Human Research Ethics Committee (ID number 178.20). Qualitative methodology using reflexive thematic analysis [43, 44], was underpinned by critical realism [45]. Critical realism focuses on real problems and acknowledges the complexities of the social world [46]. Critical realism fits with the complexity of healthcare practice to better understand the nature of the clinical work and decision making [46], hence was relevant as an overarching theory. The Consolidated criteria for reporting qualitative research (COREQ) checklist [47] was used to ensure accurate completion and reporting.

### Participants and setting

The acute hospital setting was a major trauma hospital with a 16-bed neurosurgery unit. This setting admits patients with TBI requiring neurosurgery intervention and care within the intensive care unit, high-dependency, and neurosurgery ward. Upon discharge from the acute hospital setting, patients with TBI are commonly transferred to the specialised subacute brain injury rehabilitation unit. The subacute rehabilitation setting was a specialised state-wide brain injury rehabilitation unit with 24 beds. This setting admits patients recovering from traumatic brain injury with a rehabilitative focus in preparation for discharge to the community setting. Participants included staff from both settings to gain perspectives of TBI behaviour management throughout the continuum of the TBI recovery phase in acute settings with transition to inpatient rehabilitation. Participants from the acute hospital setting provided their perspectives of barriers and enablers to managing challenging behaviours after TBI relevant to the acute setting. Participants from the subacute brain injury rehabilitation setting have expert knowledge and skills in TBI recovery, particularly behavioural rehabilitation of patients recovering from TBI [20]. Therefore, participants from the subacute brain injury rehabilitation setting were able to provide perspectives of the contextual factors (such as resources, training, environment) for improving TBI behaviour management. The perspectives of staff from both acute care and specialised TBI rehabilitation was necessary to address the study aims to elucidate the barriers, enablers and contextual factors for effective TBI behaviour management, and for opportunities to inform future recommendations for improvements in acute care. A total of 28 staff participated in four focus groups: 17 participants from the acute hospital setting, and 11 participants from the specialised inpatient brain injury rehabilitation setting. Each focus group consisted of a multi-disciplinary representation with nursing and allied health participants.

### Sampling and recruitment

Participants were purposefully recruited at each setting. Purposeful sampling is commonly used in qualitative research for the identification and selection of information-rich cases for the most effective use of limited resources [48]. Purposeful sampling involves identifying and selecting individuals or groups of individuals that are especially knowledgeable about and experienced with a phenomenon of interest [48, 49]. The availability and willingness to participate, and the ability to communicate experiences and opinions in an articulate, expressive, and reflective manner are important factors for purposeful sampling [48, 50]. A purposeful sample of relevant multi-disciplinary staff (medical, nursing, pharmacy, and allied health professionals) with experiences of working with patients with TBI in either the acute hospital setting, or specialised inpatient subacute rehabilitation setting were contacted via email, inviting staff to participate in the study. Emails inviting staff with study information were sent by the clinical leads at the acute and subacute settings who had an existing working relationship with the staff. Potential participants were informed in the emails that participation was voluntary, and their employment would not be influenced if they chose to participate or not to participate. Staff who were unable to speak or understand basic English were excluded. Once individuals had indicated a willingness to participate, they were contacted by the researcher (HB) to schedule the focus group time and place suitable to all participants.

### Implementation framework for this qualitative study

To robustly understand implementation barriers, enablers and contextual factors relating to TBI behaviour management in acute settings, this study utilised an implementation framework. Implementation science recognises multiple factors can influence translation of evidence into practice [28]. The i-PARIHS implementation framework is well recognised to support multi-disciplinary, complex interventions in acute healthcare settings [41, 51], therefore is relevant to understand implementation barriers, enablers and contextual factors and guide future strategies for improvements in care to people with challenging behaviours after TBI in the acute context. For this study, the i-PARIHS framework aligned with the complex elements of managing challenging behaviours in acute setting: ‘Innovation’ (effective TBI behaviour management); ‘Recipients’ (staff providing the care and patients receiving the innovation); and ‘Context’ (ward level and organisational characteristics and factors, and outer state, national policies and priorities). The i-PARIHS framework was adopted for the development of focus group guides, data synthesis and analysis, as outlined in Supplementary File 3. The use of the i-PARIHS framework is useful to identify barriers and enablers that influence the implementation care for TBI behaviour management in acute settings, and to guide planning with implementation strategies for future improvements to the management of challenging behaviours after TBI in acute hospital settings [41, 42].

### Data collection

Focus groups were scheduled by the study team in collaboration with shift coordinators and clinical leads to suit participant availability within clinical workloads. Due to shift changes and staffing overlap limiting participant availability, focus groups were offered as participant’s preferred method to participate. Focus groups are a commonly used qualitative technique, consisting of several participants to discuss their thoughts, experiences or perspectives on a specific topic [52]. For these reasons, and to specifically address the study aims, focus groups were the preferred method of qualitative data collection for this study. Focus groups comprised of multi-disciplinary staff to promote the overall quality and depth of information collected, with conversations between and across staff disciplines to provide deep insights and perspectives.

Focus groups were conducted from September 2021 to December 2021. Four focus groups were conducted: two at the acute hospital setting and two at the specialised inpatient brain injury rehabilitation setting. Each focus group consisted of 6–9 participants, which reflects recommended guidance on focus group sizes commonly consisting of 4–12 participants per group [53]. Previous studies have recommended more focus groups of smaller samples [53, 54], therefore four smaller focus groups were conducted rather than two larger focus groups, allowing for each participant to actively participate in discussions to gain quality and depth of information. Each focus group lasted a duration of 45–60 min. Focus groups were conducted face to face and facilitated by HB, a PhD student with clinical experience working with patients with brain injuries in the acute setting, and who had received training in qualitative research methods. HB was not part of the clinical team working with participants involved in the focus groups. Written and verbal consent was obtained from all participants following their receipt of participant information about the study. Participants did not receive compensation to participate in focus groups. Data collection ceased following completion of invitations to participate, recruitment and participation in two focus groups at two sites based on a pragmatic decision to explore the research question.

Semi-structured question guides were developed to facilitate discussion during the focus groups. The question guides were developed considering the constructs of the i-PARIHS framework to gather data reflecting barriers and enablers in effectively managing challenging behaviour after TBI in the acute hospital setting. The focus group question guides were confirmed by the study team prior to data collection, and are available in Supplementary File 1. During the focus groups, the study rationale was explained and demographic information was collected. Focus group discussions were audio-recorded and transcribed verbatim by a professional transcription service. The researcher made field notes during the focus groups to guide subsequent analysis. Transcripts were not verified by participants.

### Data analysis

Data were analysed using both inductive and deductive approaches to reflexive thematic analysis [43, 44]. Reflexive thematic analysis is well recognised for qualitative analysis of large or small data sets for data collected via interviews or focus groups; used for both inductively and deductively [44]. Table 1 outlines the six steps undertaken by the research team for reflexive thematic analysis [43, 44]. Once all focus groups were completed, transcriptions, recordings and notes were then utilised for the analysis which was carried out between May - December 2022.Transcripts and field notes were read and re-read with reflexive key ideas and comments noted. The entire focus group data set were initially inductively coded using an iterative process by first author (HB). All codes were then deductively applied to the i-PARIHS constructs of ‘Innovation’, ‘Recipients’, and ‘Context’ by two study authors (HB, SG). Coding of data were completed using Nvivo (version 12) [55]. Codes were categorised to i-PARIHS constructs to identify potential themes by authors (HB, SG, SH). Code definitions were refined and confirmed with the study team. All study authors (HB, SG, MB, SCH) were engaged in an iterative, consensus decision making process discussing the i-PARIHS constructs, codes and quotation mapping to review, define and confirm themes and subthemes. Themes were denoted as barriers or enablers as the key determinants that influence the management of challenging behaviours after TBI in the acute hospital setting. The i-PARIHS framework constructs guided the authors to understand the nuances of how and why the themes and subthemes were considered barriers and enablers. For example, a data excerpt was coded to “ward environment is overstimulating”, then deductively coded to the ‘Context’ construct of i-PARIHS. The code was then categorised to “environmental resources” characteristics based on the codebook, and subsequently themed to “Overstimulating and unsecured hospital environment” as a barrier to managing challenging behaviour after TBI in acute settings.

**Table 1:** The six steps of reflexive thematic analysis undertaken by the authors

Steps	Description
Data familiarisation	HB facilitated all focus groups face to face. HB then read and re-read transcripts for a thorough overview of the data set, noting down general and reflexive comments.
Generating initial codes	Data were systematically organised into initial inductive codes in an iterative, data-driven process by HB. Codes were assigned to sections of text relating to the management of challenging behaviours after TBI; and the factors that influencing staff providing care to patients with challenging behaviours after TBI in acute settings. The codes were then explored deductively in terms of Innovations, Recipients, and Context constructs of the i-PARIHS implementation framework by two authors (HB, SG).
Generating themes	Codes were then amalgamated into categories mapped to characteristics of the Innovation, recipients, and Context constructs of the i-PARIHS framework. Codes and categories were then reviewed by HB, SG and SCH to identify patterns and potential themes.
Reviewing themes	Once all coding, categorising and theming had been undertaken, all authors (HB, SG, SCH, MB) met as a group to discuss and review the themes. All authors reviewed the coding, categorising, and theming using an iterative, consensus decision making process. Themes were confirmed iteratively against the coded extracts (quotes) and the entire focus group data set in relation to the research question.
Defining and naming themes	All authors (HB, SG, SCH, MB) met as a group to define and name each theme, refining how the themes aid the understanding of the data. All authors reviewed the themes and subthemes, confirming the themes were accurately applied to the constructs of the i-PARIHS framework. All authors then confirmed the defined themes reflected the perspectives of staff of the management of challenging behaviours after TBI in acute settings and the findings appropriately highlighted the barriers and enablers.
Writing up	All authors (HB, SG, CSH, MB) helped with the interpretation of the results with selection of exemplar extracts for final analysis, relating the findings back to the research question. Participants did not provide feedback on the findings.

All authors were involved in interpretation and write up of the results. Participants did not provide feedback on the findings during data analysis, however a summary of results was shared with participants on completion of the study. The reflexive thematic analysis involved interpretive engagement for deep empirical exploration of data, making data saturation difficult to align, and was not the intention of the reflexive thematic analysis methods [56]. For this reason, data saturation was not examined, and findings not intended for generalisability.

### Researcher perspective

All focus groups were conducted by HB, a PhD student and occupational therapist with previous clinical experience working with patients with brain injuries in the acute setting. The research team involved in data analysis consisted of multi-disciplinary practitioners in occupational therapy (HB, SG), disability studies (MB), and psychology (SCH). Members of the research team have a broad range of knowledge and experience in clinical rehabilitation for TBI, and implementation science. All members of the research team have knowledge and experience in qualitative research methodology, undertaking a reflexive approach to openness and sensitivity to the topic to minimise personal opinions [57]. HB was a PhD student with prior relevant clinical experience in this area of practice, all authors (HB, SG, MB, SCH) contributed with a consensus-based agreement to ensure any themes were accurately supported by the data with minimal influence of researcher bias. The clinical, content and methodological experience of our research team enabled identification of data-driven themes of barriers and enablers impacting on the management of challenging behaviours after TBI in acute hospital settings from the perspectives of staff participating in the focus groups.

## Results

A total of 28 participants (17 from acute setting and 11 from subacute setting) participated in four focus groups. Acute participants had predominantly less than 10 years of experience working with patients with TBI. In contrast, a greater proportion of subacute participants were experienced with 20 or more years working with patients with TBI. Professional and experience characteristics of participants are displayed in Table 2.

**Table 2:** Characteristics of focus group participants from acute hospital and subacute rehabilitation settings

Acute hospital participants		Subacute brain injury rehabilitation participants	
Years of experience working with patients with TBI	N = 17	Years of experience working with patients with TBI	N = 11
0–5	3	0–5	1
6–15	12	6–15	5
16+	2	16+	5
**Acute hospital participants**		**Subacute brain injury rehabilitation participants**	
Professional Discipline	N = 17	Professional Discipline	N = 11
Nursing	13	Nursing	4
Allied health professional (physiotherapist, occupational therapist, speech pathologist, pharmacist)	4	Allied health professional (physiotherapist, occupational therapist, speech pathologist, social worker)	6
Medical physician or surgeon	0	Medical physician or surgeon	1

Table 3 presents the themes and subthemes for barriers and enablers staff identified to effectively managing challenging behaviours after TBI in acute hospital settings. Barriers included [1] Difficulties with clinical decision making; [2] Concerns for risks to staff and patients; [3] Hospital environment; and [4] Intensive resources are required. Enablers were [5] Experienced staff with practical skills; [6] Incorporating person-centred care; and [7] Supportive teams. Each participant has been assigned a number according to their focus group, and quotes indicate if they are from an acute or subacute setting. Coded data with quotations analysed and mapped to the constructs of the i-PARIHS framework are available in Supplementary File 2.

**Table 3:** Themes and subthemes for barriers and enablers

	i-PARIHS construct	Themes
**Barriers**	Innovation	Theme 1: Difficulties in clinical decision making
	Recipients	Theme 2: Concerns for risks to staff and patients
	Context	Theme 3: Hospital environment
	Recipients and Context	Theme 4: Intensive resources are required
**Enablers**	Recipients	Theme 5: Experienced staff with practical skills
	Recipients	Theme 6: Incorporating person-centred care Subthemes: - Understanding person centred factors - Personalised care
	Recipients	Theme 7: Supportive teams

### Barriers – difficulties in clinical decision making

Staff felt it was difficult to make clinical decisions for effective TBI behaviour management in the acute setting. Some components of TBI behaviour management (such as identifying behaviours, and medication management) were perceived as “trial and error”, due to the unpredictability of a patient’s agitated and aggressive behaviours.

*We know how to deal with someone and look at escalation but it can then just, something like that can happen, like they’re fine … and then they’re not. (FG1 acute).*

Many staff felt the medications used for settling agitated and aggressive patients with TBI in the acute ward do not work effectively, as described by one staff member: *“It doesn’t seem like the medication works sometimes.”* (FG2 acute). Staff regarded multiple factors (such as pain, hypertension, tachycardia) when considering medications for TBI behaviour management, but there was not always clarity in the clinical decision making and justification for the use of some medications.

*I think sometimes with the medications, I never quite know at what point do you give the TBI meds because you don’t want to just willy-nilly give them out because their behaviour isn’t that bad. To then it just escalates and you probably should have given them…sometimes it can be really difficult to work out what to do. (FG2 acute).*

This theme highlights the difficulties staff face in their clinical decision making when challenging behaviours can be unpredictable, and their clinical justification of when to provide pharmacological interventions.

### Barriers – concerns for risks to staff and patients

Weighing up the perceived risk versus the benefit of TBI behaviour management interventions was described as a challenge. Staff recognised that physical restraints (including mittens or shackles) should be used as a last resort option, but described concerns for their personal safety when extreme agitation and aggression was present with patients following TBI. Some staff felt the reduced use of physical restraints increased staffs’ risk of injury.

*The fact that if they’re getting agitated and you’ve used chemical restraints and nothing’s working, the reluctance to use physical restraints is still, puts staff at risk I think…I understand they’ve gone this way but I think they just need to pull it back a little bit. (FG1 acute).*

Staff described they work in an environment to care for others and if they put themselves at risk, they can get seriously hurt by aggressive patients.

*Sometimes when we have TBI patients, I wouldn’t be surprised if I got hurt at work… You come to work and you do your job. It doesn’t change how you treat them. But I just know some days I wouldn’t be surprised if I got injured at work. (FG1 acute).*

Many staff also expressed concerns for other patients on the ward who felt frightened by agitated and aggressive patients with TBI. Although staff try to reassure patients, other patients on the ward are vulnerable and often are fearful of the noise outside their room.

*It’s also other patients because sometimes they’re screaming, they’re breaking, they’re throwing things and we’ve got patients with spinal injuries that can’t move. And it’s terrifying for them. (FG2 acute).*

Staff face concerns for their own risk of injuries and the fearful responses from other patients on the ward.

### Barriers - hospital environment

Participants identified factors relating to the hospital context which impede effective TBI behaviour management. Overstimulating hospital environments with unsecured wards were clearly articulated barriers to managing challenging behaviour after TBI in the acute setting.

Adapting hospital wards or rooms to a low stimulus environment with reduced noise, low lighting, television off, limited number of visitors, and close proximity to nurses’ station enabled effective behaviour management by minimising triggers for agitation. However, the hospital environment inherently did not provide low stimulation.

*You’ve got bells going, you’ve got many teams coming in and out, lots of nurses…It’s very difficult to control that environment for those patients as well. And they pick up on noises and get distracted and that spins their behaviour then as well. (FG2 acute)*

The hospital ward where TBI patients were admitted was unsecured without a lockable door to prevent patients from absconding. For acute TBI patients with post-traumatic amnesia, who often experience confusion and disorientation, wandering and absconding from the hospital can pose a risk of injury to TBI patients, as described by one staff member: *“They can wander off to other wards, run out the hospital if they want to”* (FG1 acute).

Staff voiced concerns of the unsecured ward, and made suggestions for a locked ward, or locked section of the ward. Some staff suggested recommendations for hospital rooms specifically designed for TBI patients with low stimulation and padded walls to minimise the risk of patients hurting themselves.

*It’s keeping us safe but it’s also they’re at high risk of hurting themselves when they have a brain injury and they’re not in a protective room for themselves. (FG1 acute).*

Staff describe how the hospital environment is a contextual barrier to effectively managing patients with challenging behaviours after TBI.

### Barriers – intensive resources are required

In moments of patient’s escalating behaviours, many staff are required to intervene and settle that patient during the crisis, as described by one acute staff member: *“They might escalate again and literally have four more people assisting. So it takes a lot of the nursing staff to manage.”* (FG1 acute). The intensive resources, particularly staffing, are often not readily available.

The Code Black security team can be called to provide security and medical interventions at times of personal threat. Staff expressed their concerns in delays in Code Black team members attending to assist the ward staff in intervening with an agitated and aggressive TBI patients.

*We’ve had a situation where the patient’s being really-really aggressive so we’ve had to call a code black and then we’ve had a phone call saying they can’t come at the moment, they’re at other code blacks. What do we do? (FG1 acute).*

The lack of required staffing and specialty resources in the acute setting was described, including lack of psychology and neuropsychiatry services. The lack of neuropsychiatry services in the acute hospital setting is a barrier to effectively implementing pharmacological and non-pharmacological interventions, thereby limiting the proactive and preventative evidence-based approaches for acute TBI behaviour management. Access to neuropsychiatry was limited due to difficulties recruiting to these specialised positions, and therefore higher management approvals were required to fund private neuropsychiatrists with a waiting time in being able to attend to TBI patients at the hospital.

*That could be because [this hospital] doesn’t have their own neuropsychiatrist…We have to get it from [another hospital] and we have to get funding and it has to be approved…Seems a bit strange, doesn’t it? Like you said, it’s a major hospital (FG1 acute).*

*If we want it [neuropsychiatry] we can ask and I think sometimes source it privately but it’s difficult to get to hold of and sometimes there’s a wait. (FG2 acute).*

Staff expressed difficulty in seeking the minimum staffing resources for shifts on the ward. Casual or agency staff resourcing had been depleted since COVID-19. Some staff would often work extra shifts, resulting in less staff then available to the ward for the next day. Some casual or agency staff refused offers of shifts on the ward, not wanting to work with aggressive and agitated TBI patients.

*Before COVID and stuff when we used to get agency and stuff like that, they would refuse to come to [this ward] because being this ward is like spinal patients and it’s really heavy and then they see these behaviours (FG1 acute).*

When there are not enough staff available on the ward, or multiple staff required to manage patients with challenging behaviours, *“it takes away from the other patients as well”* (FG1 acute). Not only did staff report staffing shortages, but also that when a patient required one-on-one specialled nursing care, this came from within the ward’s allocated staffing ratio: *“Yeah we’ll get the one-on-one but that means it’s one less staff member for the rest of the ward.”* (FG1 acute). Effective TBI behaviour management is resource intensive, requiring time and staffing availability on the acute ward.

*If you want us to use less medications, less restraints all the rest of it, we need the funding to have the increased staffing that needs to come to use those strategies. (FG3 subacute).*

Many staff described the paradox of valuing good quality TBI behaviour management but lacked the funding, staffing, and resources to achieve it.

### Enablers - experienced staff with practical skills

Many staff expressed their practical experience had built their skills and knowledge of effective TBI behaviour management, as described by one acute staff member: *“experience is what you need really”* (FG1 acute).

*Education is one thing but you need to experience it… you need to experience it with someone else and that’s where the problem, because you don’t have time.”(FG1 acute).*

There were enablers to develop theoretical knowledge through education sessions and online training modules:*“our service has a series of modules, formalised training modules to support that theoretical learning component”* (FG3 subacute). However, it takes years of on-the-job practical experience to develop confidence to know how to manage challenging behaviours in this setting. Staff described the process of upskilling for practice experience *“takes years of practise”* (FG3 subacute), that it is the *“on the job learning, getting things wrong many times, then you’d learn from that”* (FG3 subacute).

*The moodles [online training courses] that we have in place, they are good to a degree, but I don’t think they give me the confidence that I feel I need to know that I’m doing it correctly. (FG2 acute).*

Staff described more education was needed on TBI behaviour management: *“the education though in this whole area is very sparse.”* (FG4 subacute). Despite the lack of education, staff were able to identify team members who have the most experience: *“we know who’s got the skills, who’s got different skills for different patients and who’s the most suitable”* (FG1 acute).

This theme highlights the importance of staffs’ practical experience to effectively manage patients with challenging behaviours after TBI in the acute setting. However, practical experience is built over years of practise with limited theoretical education currently available. Experienced skills of staff are enablers to effective TBI behaviour management, but more education and upskilling of staff in the acute clinical setting is needed.

### Enablers– incorporating person-centred care

#### Understanding person-centred factors

Factors such as opportunities for communication, responding to emotions, and responding to patient preferences in their own care are central to providing person-centred care. In individualised assessment and management of challenging behaviours after TBI, understanding the emotional factors (such as fear, anxiety, confusion) that patients may be experiencing in the acute phase of TBI can help staff understand the context of the behaviour change.

*Once I understood and framed everything around behaviour in the context of confusion and fear it makes everything more predictable, it actually becomes predictable; of course they’re behaving like that because they don’t know what’s going on and they’re scared. (FG3 subacute).*

Staff expressed challenging behaviour can be a form of communication difficulty and recommended all TBI patients with challenging behaviours should have a communication assessment.

*Just giving the person the means to communicate effectively or understanding how to communicate with them can be what manages the behaviour. (FG3 subacute).*

Staff described how understanding and responding to person-centred factors were effective approaches to managing individualised behaviour with patients with TBI.

#### Personalised care

Building rapport, trust and respect with patients and families was described as an enabler for promoting a harmonious recovery. Nursing staff were described as fundamental in identification and individualised management of the challenging behaviours.

*Nursing staff that can be key nursing staff for those people with difficult behaviours …they can get to know those people, build that rapport, figure out what some of the triggers are (FG3 subacute).*

Ensuring the "right-fit” was an important factor when allocating staff to patients to minimise personality clashes as an enabler to positive engagement in behaviour management.

*Some people just react with people differently. It’s not even just challenging, just generally really, isn’t it? Just personality clashes. You’ve got to find that right fit. (FG1 acute).*

Flexibility in how and when care is provided based on when the patient is ready to be seen was identified as personalised care, promoting patient engagement in their care and therapy.

*We’ll just go: ‘The person wants to wash now’ and then you can see everyone just sort of grabbing towels as they’re running and get them into the shower because that’s when they want to do it (FG4 subacute).*

*You might have a priority for the day of what I’m going to do that day but I know that that patient is going to be seen when they’re ready to be seen… It’s very much patient-led, and more so than other conditions I think. (FG1 acute).*

Staff described "clustering” the nursing care, rather than frequent interventions, would support patients in sleeping and minimise agitation triggers.

*Minimising the nursing interventions sometimes. If the patient is sleeping and they’re fine, they’re fine. Just leave them alone.… Cluster care. (FG1 acute).*

These findings highlight that incorporating person-centred care as fundamental enablers for effective TBI behaviour management. Efforts to understand person-centred factors, building rapport, with personalised care delivered in a flexible way are enabling approaches with patients with challenging behaviours after acute TBI.

### Enablers – supportive teams

A cohesive, supportive team enabled the development of skills and knowledge. More experienced staff provided peer-support to their less experienced colleagues when the context and time allowed. Staff described effective TBI behaviour management as a whole team approach. When staff reported feeling overwhelmed by their patients with constant challenging behaviours, regular rest breaks and debriefing with peers were described as strategies to maintain resilience.

*You really can’t manage behaviour effectively without the input from the whole team because there’s so many things that can impact what’s going on with a person’s behaviour. (FG3 subacute).*

Although the lack of neuropsychiatry services in the acute hospital was described as a barrier, on the occasions when neuropsychiatrists were able to consult on the acute ward, staff described how their recommendations made a prompt and positive impact on the challenging behaviours. Staff also expressed they had learned a lot when specialist services consult with the team.

*What we’ve found is the sooner neuropsychiatry come in and change different medications actually some of the agitation calms right down (FG1 acute).*

*We’ve had the psychiatrists, psychiatry and psychology sit down and explain what part of the brain’s been damaged and how this effects behaviour and what’s happening. (FG3 subacute).*

Staff expressed how important it was to feel supported by leadership. As a team, staff felt their peers had a good understanding of the emotional and physical toll of working with patients with TBI.

*You’ve got to have a strong team who understand this and know when to step in and step out; it does help. It helps when your manager says how are you feeling, are you okay? (FG3 subacute).*

Feeling supported for their roles from the leadership staff was an important factor in feeling valued, cared for and in promoting resilience. Additionally, staff felt appreciated by families and patients.

*Recent families that we’ve had or patients have had like thank you’s and appreciation from the patient’s families – I guess that’s most recently how I feel that that has been valued because they’ve been very thankful of our input/education and providing resources/reassurance (FG2 acute).*

A cohesive, supportive team, working together and feeling valued by leaders were enablers for opportunities to deliver effective TBI behaviour management in the acute setting.

## Discussion

Staff expressed several barriers and enablers related to the management of challenging behaviours after TBI in the acute setting. By adopting the i-PARIHS framework, staff perspectives to understand the innovation, recipient and context related barriers and enablers to managing challenging behaviours after TBI in the acute hospital setting were examined.

The barrier of difficulties with clinical decision making relating to TBI behaviour management relevant to the acute setting was illustrated by staff describing uncertainty and unpredictability in decisions for clinical interventions, describing “trial and error” approaches to behaviour management. Staff were concerned for their risk of injury, and faced challenges in justifying the perceived benefits and consequences of behaviour management interventions (for example, reducing physical restraints and risk of injuries). These findings support the need for more evidence and guidelines to support management interventions for TBI challenging behaviours in acute settings [12–14]. Clinical practice guidelines outline recommendations for the management of challenging behaviours after TBI relevant to acute settings [15, 16, 19, 23], but few guidelines provide detail on how to implement recommendations into clinical practice [19]. Therefore, gaps in guidelines implemented in practice limits clinical decision making for staff working with TBI patients with challenging behaviours [19, 58].

The hospital environment can be overstimulating and unsecured, thereby increasing triggers for agitation. Staffing shortages, lack of specialised services, and reduced staffing ratios were emphasised by staff as a barrier to the required resources to enable effective and quality TBI behaviour management in the acute setting. The findings of this study, align with previous research, emphasising that limited staffing, and inadequate resources are barriers in providing care to patients with TBI in the acute setting [27, 31, 40].

Despite the significant contextual barriers present in acute hospital settings, results from this study identified enablers to effective TBI behaviour management in the acute setting.

Staff with practical experience and skills are fundamental in effectively managing challenging behaviours after TBI in the acute setting. However, upskilling takes time with prolonged practical experience integral to skill development. Clinical staff often learn skills for managing challenging behaviours through “on-the-job” learning [27]. Formal training and education in this area of practice is scarce, emphasising the need for more frequent and formal training programs to develop and maintain staffs’ skills in managing challenging behaviours after TBI within the acute hospital setting [27, 31, 40].

Providing personalised care, with an understanding of communication, emotional and personal factors were enablers, and are key components of pro-active, individualised, person-centred care for positive behaviour principles for people with challenging behaviours after TBI in community settings [59, 60]. Supportive, cohesive teams who were valued by their leaders are positive enablers in effective TBI behaviour management.

There are few studies that have identified barriers and enablers relating to TBI challenging behaviour management within the acute hospital context from the perspectives of multi-disciplinary staff [40]. Novel findings have been identified, emphasising enablers to effectively managing challenging behaviours after TBI in the acute hospital setting. Furthermore, with a lack of studies investigating the management of challenging behaviours after TBI in acute settings incorporating implementation frameworks, this study adds new knowledge. These findings are imperative in providing novel, pre-implementation understandings for initiatives that aim to improve the management of challenging behaviours after acute TBI. A concurrent study is being undertaken by the researchers to explore the experiences of families of patients who experienced challenging behaviours in the acute setting after TBI for further opportunities for improvements to acute care incorporating the perspectives from families.

Future improvements can be trialled using implementation strategies to address the identified barriers and leverage the enablers to increase the likelihood of sustained implementation of effective TBI behaviour management in acute hospital settings [32]. Following the identification of barriers and enablers highlighted from this study, implementation strategies can be developed and tested for sustained adoption and implementation of improvements into acute clinical practice [58, 61]. By understanding the contextual factors relating to the ‘Innovation’, ‘Recipient’ and ‘Context’ constructs of the i-PARIHS framework, future implementation strategies operationalised through tailored facilitation can adapt and sustain change for improvements in care for people with challenging behaviours after TBI relevant to the acute context [41, 62]. As evident in previous research utilising i-PARIHS to implement complex interventions in practice [62–66], implementation strategies for improvements to TBI behaviour management in acute care will require multifaceted system wide changes to address gaps in innovation characteristics, guideline development, policy environment, management support, stakeholder engagement, training programs, resources and tools with organisational and local facilitation, evaluation and tailoring to the context of the acute hospital setting [62].

This study utilised a robust qualitative methodology underpinned by an implementation science framework, thus demonstrating several strengths. However, some limitations need to be acknowledged. There were differing number of participants and mix of professionals within the acute and subacute focus groups, however all participants contributed to discussions. No acute medical physicians participated in the focus groups, despite invitation to participate. Within one focus group, a ward-based manager was present which could have influenced other staff disclosing concerns and barriers relating to workforce, workload and leadership support. This study was conducted during COVID-19 pandemic whereby the impact of reduced workforce and hospital demand could have influenced participant’s perspective on barriers and difficulties in delivery of care.

## Conclusions

This qualitative study identified the barriers to managing challenging behaviours after TBI in acute hospital settings relate to the difficulties with clinical decision making, concerns for risks of injury, challenges within the hospital environment, and lack of resources required, particularly staffing workforce. Experienced staff are fundamental, however upskilling takes time, with more education needed. Improvements to TBI behaviour management in acute care will require developed, trialled and tailored implementation strategies for multifaceted system wide changes to address barriers, and leverage individualised, person-centred care from a whole team approach.

### Electronic supplementary material

Below is the link to the electronic supplementary material.

Supplementary Material 1

Supplementary Material 2

Supplementary Material 3

## Data Availability

Data supporting the results, including question guides, analysed coded data and quotations mapped to the constructs of the i-PARIHS framework are available in supplementary files.

## Abbreviations

COREQ: Consolidated criteria for reporting qualitative research
i-PARIHS: Integrated-Promoting Action on Research Implementation in Healthcare Settings
TBI: Traumatic brain injury
