# Supplementary material for: Barriers and enablers to managing challenging behaviours after traumatic brain injury in the acute hospital setting: a qualitative study
Source: BMC Health Serv Res. 2023 Nov 16;23:1266. doi: 10.1186/s12913-023-10279-z (PMC10655469; doi:10.1186/s12913-023-10279-z)
Supplement: Supplementary file 1 — Supplementary Material 1 [file 12913_2023_10279_MOESM1_ESM.pdf]

Supplementary File 1. Focus group question guide for acute and subacute settings

Acute Focus Group Guide

Focus Group Demographic Survey

1) What is your professional discipline?
2) How many years have you worked with patients with TBI?
3) Which setting do you work at?
   a) Acute setting
   b) Subacute Brain Injury rehabilitation setting

i-PARIHS Construct	*Question*
**Recipients**<br><br>- Skills<br>- Knowledge<br>- Decision making	**1) What is your perspective on your current level of knowledge and confidence related to TBI behaviour management?**<br>a) Prompt: Is there available knowledge/learning opportunities about effective management of challenging behaviours in your setting? If yes, what are these?<br>b) Prompt: Can you describe any barriers to learning the practical skills of how to manage challenging behaviours with patients with TBI? If yes, what are these?<br><br>**2) How easy or difficult do you find coming to a clinical decision about TBI behaviour management?**<br>a) Prompt: How do you identify if a patient needs management of challenging behaviours?<br><br>**3) Do your emotions (such as fear) ever influence your management of challenging behaviours in patients with TBI?**<br>a) Prompt: What strategies could improve this?<br>b) Prompt: How do you maintain resilience?
**Evidence/Innovation**<br><br>- Evidence<br>- Relevance to setting	**4) Current evidence recommends TBI behaviour management involves comprehensive behaviour assessment, commence with non-pharmacological approaches, then progressing to pharmacological approaches. Evidence and policy recommend reducing use of restraints (including physical, mechanical, and chemical). Beta Blockers for acute agitation. Positive behaviour approaches in rehabilitation. Do you think these**

	**recommendations are practical/realistic in your setting? Why/why not?**<br>a) Prompt: How could TBI behaviour management be improved?
**Local Context**<br>- Leadership<br>- Culture<br>- Staff valued<br>- Environment and resources	**5) How does the physical environment influence effective TBI behaviour management?**<br>a) Does the physical environment in the acute setting help or hinder effective management of challenging behaviours? How?<br>b) Do you have suggestions on how the physical environment can be improved in hospital settings?<br><br>**6) What are the necessary resources to effectively managing challenging behaviours for patients with TBI?**<br>a) Prompt: available staffing, access to doctors to prescribe appropriate medications, assessments used, forms, behaviour support plans, therapy<br><br>**7) How is your role in managing challenging behaviours in patients with TBI valued in your setting? How is this demonstrated?**
**Organisational and Outer Context**<br>- Strategic priorities<br>- Policies & procedures<br>- Systems<br>- Teamwork	**8) What effect do colleagues and management team have on effective TBI behaviour management? How?**<br><br>**9) What workforce factors influence effective TBI behaviour management in your setting?**<br>a) Prompt: rostering, staffing numbers, stable teams, rotating staff, agency/casual v's regular staffing

Subacute Rehabilitation Focus Group Guide

Focus Group Demographic Survey

1. What is your professional discipline?
2. How many years have you worked with patients with TBI?
3. Which setting do you work at?
   a) Acute setting
   b) Subacute Brain Injury rehabilitation setting

i-PARIHS Construct	*Question*
**Recipients**<br>- Skills<br>- Knowledge<br>- Decision making	**1) What has helped develop your knowledge/learning about effective TBI behaviour management in your setting?**<br><br>**Prompt**: How are the specialised/specific TBI management approaches taught and applied to patients with TBI?<br>Prompt: Acute staff report skills are learnt through experience and trial and error. How do you find this in developing skills in TBI behaviour management in the subacute setting?<br><br>**2) Do your emotions (such as fear) ever influence your management of challenging behaviours in patients with TBI?**<br>a) Prompt: What strategies could improve this?<br>b) How do you maintain resilience?
**Evidence/Innovation**<br>- Evidence<br>- Relevance to setting	**3) Current evidence recommends TBI behaviour management involves comprehensive behaviour assessment, commence with non-pharmacological approaches, then progressing to pharmacological approaches. Evidence and policy recommend reducing use of restraints (including physical, mechanical, and chemical). Positive behaviour approaches in rehabilitation.**<br>Do you think these recommendations are practical/realistic in the acute hospital setting? Why/why not?<br><br>**4) What could be implemented in acute hospitals to improve management of challenging behaviours in the early phase of TBI recovery?**
**Local Context**<br>- Leadership<br>- Culture<br>- Staff valued<br>- Environment and resources	**5) How does the physical environment influence effective TBI behaviour management?**<br>a) Prompts: Do you have suggestions on how this can be improved in hospital settings?<br><br>**6) What are the necessary resources required to effectively manage challenging behaviours for patients with TBI?**<br>a) Prompt: available staffing, access to doctors to prescribe appropriate medications, assessments used, forms, behaviour support plans, therapy<br><br>**7) How is your role in managing challenging behaviours with patients with TBI valued in your setting? How is this demonstrated?**
**Organisational and Outer Context**	**8) What effect do colleagues and management team have on effective TBI behaviour management? How?**

- Strategic priorities<br>- Policies & procedures<br>- Systems<br>- Teamwork	
