# Supplementary material for: Barriers and enablers to managing challenging behaviours after traumatic brain injury in the acute hospital setting: a qualitative study
Source: BMC Health Serv Res. 2023 Nov 16;23:1266. doi: 10.1186/s12913-023-10279-z (PMC10655469; doi:10.1186/s12913-023-10279-z)
Supplement: Supplementary file 2 — Supplementary Material 2 [file 12913_2023_10279_MOESM2_ESM.pdf]

Supplementary File 2. Codes and quotes categorised to i-PARIHS

i-PARIHS Construct - INNOVATION		
**Category**	**Code**	**Quotes**
**Compatibility**	Comprehensive behaviour assessment is difficult in acute settings	"the comprehensive assessments they can be looking at, assessing a patient at a time and they're trying to identify their triggers and the warning signs but how easy or hard is that to do with this patient if they come from ICU and they've been sedated" (FG1 acute)<br>"But if there was behaviour assessments to start with and non-pharmacological approach or sooner chemical restraint... The whole thing works together otherwise little things on their own don't work" (FG1 acute)<br>"getting someone to fill that out is really hard if nobody knows about it, because I can't spend all day doing it" (FG1 acute)<br>"Little bits, you just do an assessment, just do non-pharma[cological]…You can't just do one of those things, you have to do all of them." (FG2 acute)<br>"I think getting that comprehensive assessment is where it's harder… to start with because if you haven't got that you can't do the next step." (FG3 subacute)
**Compatibility**	Behaviour management is limited by unpredictability.	"Initially when he came he just slept the whole day and two or three days later he was, he pushed the whole, picked up a

		whole cupboard and tipped it over, smashed the computer where the nurse was studying, sitting at their computer so you don’t always know what to expect” (FG1 acute)<br>“we know how to deal with someone and look at escalation but it can then just, something like that can happen, like they’re fine and then they suddenly flip out or they’re very asleep… and then they’re not.” (FG1 acute)
**Compatibility**	TBI behaviour management can be trial and error	“The pressure you feel about managing or following maybe what the best practice might say versus what helps manage their behaviour, can sometimes be conflicting.” (FG2 acute)
**Understanding person-centred factors**	Care is flexible based on when the patient is ready to be seen	“you might have a priority for the day of what I’m going to do that day but I know that that patient is going to be seen when they’re ready to be seen… It's very much patient-led, and more so than other conditions I think.” (FG1 acute)<br>“everyone’s an individual. So to get the best out of them we have to get to know them and you just don’t have time for that in acute.” (FG4 subacute)<br>“We’ll just go the person wants to wash now and then you can see everyone just sort of grabbing towels as they’re running and get them into the shower because that’s when they want to do it” (FG4 subacute)

**Understanding person-centred factors**	Building respect for harmonious rapport	"a lot of it is gaining their trust and just showing respect really." (FG3 subacute)<br>"Initially there's a lot of work that you've got to do for the patient, for the family to have a little harmonious ride through rehab." (FG3 subacute)<br>"With the families, just because they're here don't shut them out; you've got to draw them in because they are part of it" (FG3 subacute)
**Understanding person-centred factors**	Challenging behaviours are a form of communication deficit	"We know behaviour is a communication act and I think what we often notice here is that sometimes just giving the person the means to communicate effectively or understanding how to communicate with them can be what manages the behaviour." (FG3 subacute)<br>"I think also use of interpreters because that doesn't often get done as much as it should in acute." (FG3 subacute)
**Understanding person-centred factors**	Challenging behaviour can come from fear and confusion	"People are scared, they're in this bed, their life's been taken away from them, they're scared, their families are scared, so they behave differently and there is agitation and this fear." (FG3 subacute)<br>"Once I understood and framed everything around behaviour in the context of confusion and fear it makes everything more predictable, it actually becomes predictable; of

		course they're behaving like that because they don't know what's going on and they're scared." (FG3 subacute)
**Research evidence**<br>**Clinical experience**<br>**Patient needs**	Restraints are the last resort	"We try not to use them as much as we can, obviously unless it's, patient safety or staff safety issue." (FG2 acute)<br>"It's always a, obviously the last resort." (FG2 acute)<br>"Physical restraints are very-very last resort and even in extreme cases where it is unsafe, when we have patients that do, are being physically and verbally aggressive, sometimes we probably don't go there, we probably should be interfering before someone gets hurt." (FG1 acute)
**Compatibility**	Reduced use of restraints has increased risk of staff injuries	"The patient is aggressive, verbally abusive and so that doesn't warrant restraints and then they're becoming more and more agitated but no-no we can't use any sort of restraints and then it's too late. Nurse gets hit." (FG1 acute)<br>"The fact that if they're getting agitated and you've used chemical restraints and nothing's working, the reluctance to use physical restraints is still, puts staff at risk I think. I just, I understand they've gone this way but I think they just need to pull it back a little bit." (FG1 acute)
**Compatibility**	Medications do not always work effectively	"So chemical restraints we use those but often they don't do anything, a patient's too far gone." (FG1 acute)<br>"And then that's trial and error for the ward then basically." (FG2 acute)

		"Sometimes you just can't control it pharmacologically" (FG1 acute)<br>"It doesn't seem like the medication works sometimes." (FG2 acute)
**Compatibility**	Balancing sedation and agitated behaviours	"I think sometimes with the medications, I never quite know at what point do you give the TBI meds because you don't want to just willy-nilly give them out because their behaviour isn't that bad. To then it just escalates and you probably should have given them…sometimes it can be really difficult to work out what to do." (FG2 acute)
**Clinical experience**	Restraints are used, need to be recognised and justified	"We use restraints every day, we do. Automatically we use restraints. But as long as we know what we're using, we know why we're using them and you know, they are, well I suppose you'd say reasonable." (FG4 subacute)<br>"Again as long as we know what we're doing and we've got reasons why we're doing it for safety because that's the overriding thing, that's okay. But it's just making sure that we recognise that we use restraint every day, it's just degrees of it." (FG4 subacute)
**Compatibility**<br>**Clinical experience**	Fine line between behaviour management and safety	"It's fine line between keeping the patient safe and keeping us, nurses and other people safe" (FG1 acute)<br>"It's all just a balance because they're so unpredictable, trying not to be too in their face but and given the space. But then

		not go too far because you don't want them to hurt themselves so or us. So I don't know, it's all a balance." (FG2 acute)<br>"They're very aggressive, the time it takes for them to have an effective medication it's very unsafe" (FG1 acute)
**Clinician experience**<br>**Personal attributes of staff and team – recipients**	Chemical restraint is not often thought of as a form of restraint	"You definitely use chemical over physical restraint." (FG1 acute)<br>"I guess chemical restraints probably aren't thought of as a restraint system." (FG2 acute)<br>"it's restraint, but it's not visible." (FG2 acute)<br>"there's a bit of a stigma around physical restraints; whereas chemical restraints are an easier thing to get, there's less paperwork." (FG2 acute)<br>"It may be perceived as helping the patient rather than harming. Because you're seeing it as a treatment as well, so you're managing the behaviours… managing their safety and your own safety as well, so it's not directly seen as a restraint as just a physical restraint would be seen" (FG2 acute)
**Clinical experience**<br>**Compatibility**	Multiple factors to consider when using medications (not clear cut)	"I think the use of medications is an interesting one because a lot of times you're using some of the you know, a lot of the yeah, if they're tachycardiac and hypertensive … beta blocker. And you may not do it primarily for behaviour but you'll know that it will have an effect on behaviour. So

		you'll often be using medications for a long list of reasons and if you're going to choose it for one thing you will bear in mind that it may have effect on something else." (FG4 subacute)<br>"So I don't think it's as simple as saying non-pharmacological first. It's not ever quite that clear cut. And because I think … TBI they're often pretty sick." (FG4 subacute)
**Research evidence**<br>**Clinician experience**	Pharmacological management is started at a low level	"if you have to go pharmacological management, going through all of them, they're less, we start less and then work your way up." (FG2 acute)<br>"Start with a low level of pharmacological management." (FG2 acute)
**Patient experience**	More education is needed to families in the early stage	"I don't know if there's a lot of information or education around what happens next after someone's survived." (FG4 subacute)<br>"that education just equally to the family as well just because they just want to help. So if they know they can help more by doing something else they'll, most families will take it on board and run with it." (FG4 subacute)<br>"But that education earlier on would really help" (FG4 subacute)<br>"A good one page would be good for the relatives because again also I think they find it overwhelming. Really just all relatives, because I find I'm repeating myself" (FG2 acute)

i-PARIHS Construct - RECIPIENTS		
**Category**	**Code**	**Quotes**
**Teamwork**<br>**Skills and knowledge**	Staff develop skills through peer support from experienced colleagues	"Teach people but then you've got to have time to do that" (FG1 acute)<br>"we want them to get the experience with that patient so that they can be, in the future, someone who we can say they've got that experience." (FG1 acute)<br>"if I'm feeling really stuck and I'm like this is not working I might go can I do a joint [session] with you so I can see what you're doing and you've got better rapport so let's just see what you're doing and what I can take on board." (FG3 subacute)
**Teamwork**	Relieving 1:1 staff are supported by regular staff where possible	"We try and, if it is a reliever that has the one to one, we try and rotate and take half a shift each, if it's a really aggressive patient. So they don't have the whole issues for eight hours straight. But it doesn't always work at the moment. And we do have a lot of relievers and agencies." (FG1 acute)
**Teamwork**	Agitated TBI patients don't get as much therapy from allied health staff	"In Allied Health as opposed to nursing staff is that if someone is displaying a lot of those behaviours, we wouldn't see them that day." (FG1 acute)<br>"if I see there's a patient and I've looked on the notes and I can see they're having a bad day. I might avoid seeing them that day" (FG2 acute)<br>"Also they won't get the same level of input that a patient that was more calm might get, because I've seen they've been really up and down.

		I don't want to set them off, I know how it's hard it's been to settle them all day." (FG2 acute)<br>"in your head, they're too agitated, they won't really benefit from what I'm going to do. But actually maybe they're sort of on that cusp of it would actually be really beneficial for them to be seen." (FG2 acute)
**Teamwork**	Effective behaviour management is a whole team approach	"you really can't manage behaviour effectively without the input from the whole team because there's so many things that can impact what's going on with a person's behaviour." (FG3 subacute)<br>"coming together as a team to formulate somebody's plan and then having that same conversation" (FG4 subacute)
**Skills and knowledge**	Staff know who has experience in this area of practice	"We probably know who's got skills, who's got different skills for different patients and who's the most suitable" (FG1 acute)
**Skills and knowledge**	Experience builds knowledge and skills	"experience is what you need, really. You can teach people all the things to do with some of the challenging behaviours, but you've got to be confident enough to be able to apply that when you're with patient and they are starting to go a bit challenging" (FG1 acute)<br>"So education is one thing but they just need, you sort of need to experience it. But you need to experience it with someone else and that's where the problem … because you don't have time. Because you're talking about someone new specialling them … but someone would have to be with them to help them." (FG1 acute)

		"So education is one thing but they just need, you sort of need to experience it." (FG1 acute)<br>"so it was that on the job learning, getting things wrong many times, then you'd certainly learn from that." (FG3 subacute)<br>"I think it takes years of practise" (FG3 subacute)<br>"there's a really big difference of actually knowing and doing" (FG3 subacute)<br>"seeing previous behaviours and being able to apply approaches that we have in place then with that client with another client" (FG4 subacute)
**Skills and knowledge**<br>**Personal attributes of staff and team**	It's the brain injury that causes the behaviour	"We know it's their brain injury and not them." (FG1 acute)<br>"I've had relievers who just think what's wrong with this person. I'm not looking after this person. They don't realise it's not them. It's the injury. So that just comes with experience." (FG1 acute)<br>"I turn it back to okay, how is this related to their brain injury, this is not a personal thing, this is a fascinating clinical dilemma or question that I as a therapist have the skills and knowledge to unpack, explore and treat." (FG3 subacute)
**Resources**<br>**Skills and knowledge**	Online training moodles for theoretical learning	"I think currently it would be moodles that we have in place, and they are good to a degree, but I don't think they give me to confidence that I feel I need to know that I'm doing it correctly." (FG2 acute)<br>"our service has a series of modules, formalised training modules to support that theoretical learning component" (FG3 subacute)

**Skills and knowledge**	More education needed on behaviour management	"I still think to be perfectly honest, the education though in this whole area is very sparse." (FG4 subacute)<br>"I really worry about some of the new staff that don't get any training. They literally get thrown into it and they kind of go sink or swim." (FG4 subacute)<br>"if you go to build your knowledge you actually need to build it from medical school or nursing school. They need to have more of it" (FG4 subacute)<br>"The [registered nursing] course was nothing to do with it. There was nothing about it." (FG1 acute)
**Resources**<br>**Skills and knowledge**	Not enough resources in acute setting for effective behaviour support principles	"understanding the function of behaviour and I know – well my basic understanding of that comes back to positive behaviour support principles, but what I'd really love to see is more of that in the acute setting but I just don't know what the solution is, I just don't think there's enough resources or knowledge" (FG3 subacute)<br>"In acute when we've had very much escalated behaviour and literally there's no resources …it gets to a point where literally it is four point restraints and chemical restraints and what not, it just gets to that point" (FG4 subacute)<br>"we don't have anything apart from what's online, on the intranet saying protocols. We don't have anybody specific come in for [positive behaviour] support" (FG2 acute)

**Resources**	Consulting services for behaviour management in the acute setting	“That is part of our role as a state-wide service is to provide education and consulting” (FG3 subacute)<br>But they have a behaviour team, you know they have a trache team and they have other teams that review… They have a consulting team yeah, that does behaviour rounds.” (FG3 subacute)<br>“it’s like a fire warden, behaviour support warden type person like in terms of you know, taking a semi-sense of responsibility to be able to you know, be the person to go to or provide some training” (FG4 subacute)
**Resources**<br>**Skills and knowledge**	Staff learn from psychiatry and psychology peers	“we’ve had the psychiatrists, psychiatry and psychology sit down and explain what part of the brains been damaged and how this effects behaviour and what’s happening.” (FG3 subacute)<br>“I’ve certainly learned a lot from some of the complex ones because we’ll then sometimes have a team will get together and it’ll be like mixed like interdisciplinary with allied health, nursing, some of the doctors will be there as well. And we’ll be trying to work out sort of a behavioural plan and we’ll have a small group with a psychologist and things trying to work out a positive behavioural plan.” (FG4 subacute)
**Skills and knowledge**	Security guard’s skills in de-escalation are variable	“Some guards are so good and so experienced and really help in the de-escalation.” (FG1 acute)

		"some that have the experience, they just know and then others actually escalate the problem because they don't know how to deal appropriately with them. They just wind them up more." (FG1 acute)<br>"Security did agitate that patient and it was very focused on security and it ended up the aggression was directed at security." (FG1 acute)<br>"I think with the security guards, they need a little bit of education too. I know it's not in their role description." (FG1 acute)<br>"Variable. Every now and again you'll get one who's really quite, gets quite on the wavelength but I think that's more luck." (FG4 subacute)
**Personal attributes of staff and team**	Staff feel concerned for their own safety	"sometimes when we have TBI patients, I wouldn't be surprised if I got hurt at work… You come to work and you do your job. It doesn't change how you treat them. But I just know some days I wouldn't be surprised if I got injured at work." (FG1 acute)<br>"you might not get my best self because I'm just distracted by thinking about my safety. So also the quality of care might not be as good because I'm distracted by that as well." (FG2 acute)<br>"we work in an environment you want to help people, and they … go I want to help them, I'm going to help them, they're anxious or whatever. And but if you put yourself at risk and that's the problem, you can get seriously hurt." (FG4 subacute)
**Personal attributes of staff and team**	Other patients are fearful of aggressive patients	"it's not just our emotions but the emotions of the other patients, and they've expressed that they're very frightened of this person and we have to look after them as well. It can be quite difficult." (FG1 acute)

		"it's also other patients because sometimes they're screaming, they're breaking, they're throwing things and we've got patients with spinal injuries that can't move. And it's terrifying for them." (FG2 acute)<br>"often the nurses are trying to protect the TBI patient from themselves but also protect the rest of the ward." (FG2 acute)<br>"our patients are very vulnerable, so they're often quite fearful of the noise that's outside their room or somebody opening a door as well, so." (FG4 subacute)
**Personal attributes of staff and team**	Emotions don't influence clinical decisions	"You're more likely to give medication but that's just a logical step. I don't think it's a fear based or emotion based response. It's just a logical response to the situation." (FG1 acute)<br>"I don't think my emotions would influence calling a Code Black but more the experience." (FG2 acute)
**Personal attributes of staff and team**	Ensure the right fit between staff and patient	"staff, security, the patient, they're all – everyone's got their own little way of doing things and being challenging themselves. Some are really good combinations and some you think are not a good combination." (FG1 acute)<br>"Some people just react with people differently. It's not even just challenging, just generally really, isn't it? Just personality clashes. You've got to find that right fit." (FG1 acute)<br>"so you try to match that person's needs as much as you can, but at the end of the day sometimes you need a different therapist" (FG3 subacute)

		"we need to think about allocating appropriately, giving you a bit of a rest or is this the right match with this patient because our nursing workforce is variable," (FG3 subacute)
**Personal attributes of staff and team**	Staff feel exhausted emotionally	"you see …when you have a lot of them it's a bit much, isn't it really, the whole time. It's hard work and it's mentally draining." (FG1 acute)<br>"when we had multiple (TBI patient's) back to back. It was just very fatiguing, very mentally draining and you can spend a whole shift dealing with that one person and not really much else." (FG1 acute)<br>"This is a very difficult patient group and it's experience… to go through this and think about it and reflect on it." (FG1 acute)<br>"From an emotional point of view, it takes its toll a little bit" (FG2 acute)<br>"So sometimes it can be hard to, to want to look after them for that reason…You just knew your day already sort of thing and it was just exhausting emotionally." (FG1 acute)<br>"It doesn't matter how resilient you are, how could you … to continue that day-to-day it's almost, it is a bit beyond." (FG4 subacute)<br>"that was a bit of a catch 22 in the acute because again, their knowledge and skills are built from experience and so the more experienced nursing staff were often allocated to people with brain injury, but then often after a week of specialling they'd feel fatigued and exhausted and burnt out and scared, but then the next day get a casual cover or someone and then they still have to provide a lot of support

		because the casuals don't know how to manage it. And so it just sounded like this vicious cycle of not being able to manage." (FG3 subacute)
**Resources**	A cheat sheet to guide staff on management strategies	"if you'd had a cheat sheet or something. That you can quickly refer to because we do it, it's mandatory but if you're not practicing it daily you don't always remember." (FG2 acute)
**Resources**	Clinical protocols can guide and justify treatment	"we've got that streamlined TBI protocol with the pharmacology like protocol attached with the non-pharmacological. That really helps with clinical decision making…it guides you to treatment options and management. It does help justify the treatment and management of a patient." (FG1 acute)<br>"the protocols are okay but it doesn't really help in the second by second management which can quickly escalate." (FG1 acute)
**Resources**	Multiple nursing staff required to manage a patient with escalating behaviours	"if we have someone very aggressive or agitated, it takes more than that one person to … two, three, four people with that patient. At that time when they escalate." (FG1 acute)<br>"They might escalate again and literally have four more people assisting. So it takes a lot of the nursing staff to manage." (FG1 acute)<br>"It takes away from the other patients as well." (FG1 acute)<br>"But sometimes we end up with 2 security guards and a nurse if they were that violent" (FG2 acute)

**Maintaining resilience**	Debriefing and team support helps staff maintain resilience	"all we can do is support them and like you say, say it's normal to feel upset and feel overwhelmed or anxious about this patient." (FG1 acute)<br>"we all had a debrief the next day, and with the patients that were still there as well" (FG1 acute)<br>"Sometimes on a Friday at the end of the week with whoever's on we'd meet up and debrief at the pub." (FG2 acute)<br>"we've got a culture I think of being able to have a bit of humour and laugh things off and I think a culture of not judging other people if they feel scared of someone's behaviour" (FG3 subacute)<br>"there's a sort of a lot of informal debriefing that you know, that as a manager probably wouldn't want to hear, but it's therapeutic to a degree." (FG4 subacute)
**Maintaining resilience**	Nursing staff need regular rest breaks when behaviours are constant	"This is it, we were going to – I can't do this much longer, I need a break" (FG3 subacute)<br>"And we do have breaks and we have regular if the behaviour is non stop, it's too much sometimes eight hours with that one person." (FG3 subacute)
**Maintaining resilience - recipients**<br>**Feeling valued - context**	Staff feel valued by patients and families	"The families are normally good generally …It is nice for them to come back and say hello" (FG1 acute)<br>"recent families that we've had or patients have had like thank you's and appreciation from the patient's families – I guess that's most recently how I feel that that has been valued because they've been very thankful of our input/education and providing resources/reassurance" (FG2 acute)

		"I think the families are quite appreciative of not just allied health, but of everyone" (FG2 acute)

i-PARIHS Construct - CONTEXT		
**Category**	**Code**	**Quotes**
**Environmental resources**	The ward environment can be overstimulating	"It's the noise, you can't keep them in a quiet environment, it's the light… An unfamiliar environment." (FG1 acute)<br>"you've got bells going, you've got many teams coming in and out lots of nurses. So it's and other patients as well. So it's very difficult to control that environment for those patients as well. And they get pick up on noises and get distracted and that spins their behaviour then as well." (FG2 acute)<br>"And more access to the right environments and more access to side rooms where we can provide that low stimulus environment and in an ideal world having TBI specific rooms where there could be environmental prompts or environmental things that are of meaning to each patient that would make them more calm or whatever their calming or their antidotes their triggers were would help, whether it's music or having some sort of environmental cues to help." (FG2 acute)<br>"It's probably our biggest issue is that we can't find that quiet isolated area where it's recommended for patients to have that low stimulus… And it's noisy, lots of staff, lots of teams coming in and out, telephone

		ringing all the time, visitors in and out, patients being admitted all day and night, so it's very difficult to be quiet calm time." (FG2 acute)<br>"Because I know with the acute you have multiple people knocking on the door several times and then the … overload when the noise is constant and the lights has to be turned on and etcetera, the environment itself it's constant. (FG4 subacute)
**Environmental resources**	Behaviour management involves low stimulation environment	"we have the side room with low stimulation" (FG1 acute)<br>"to prevent escalation of behaviours is like reduce the noise, turn the lights down, all those kind of things" (FG2 acute)<br>"We remove the phone, we just put the TV out the way, we don't encourage that. There's normally blinds are shut at night." (FG2 acute)<br>"And try and minimise the amount of people … as well and also no use of a mobile phone either." (FG2 acute)<br>"We try and follow the normal lighting patterns of the day, like each morning open it somewhat, but if they're resting you have it dark" (FG2 acute)
**Environmental resources**	Position patients close to the nurse's station	"We try and put the patients near the nurses station as well, just so that there's always somebody – well hopefully there's always somebody around that can help if things don't go so well." (FG2 acute)<br>"but just close to the nurses' stations for falls regular toileting stuff like that." (FG2 acute)
**Environmental resources**	A locked ward may prevent patients absconding	"They can wander off to other wards, run out the hospital if they want to" (FG1 acute)

		"They can literally just run out into the street." (FG1 acute)<br>"it's about keeping them close to us where we have the staff in that area, not being out in the corridor, not being outside the hospital premises. At least we can deal with it in the unit" (FG1 acute)<br>"A locked ward…Or even a section of the ward could be like a TBI area, a section." (FG1 acute)<br>"And locked, you know lock the door so that they can't take off too far." (FG2 acute)
**Environmental resources**	Padded rooms may prevent aggressive patients hurting themselves	"it's keeping us safe but it's also they're at high risk of hurting themselves when they have a brain injury and they're not in a protective room for themselves." (FG1 acute)<br>"And padded as well so they don't harm themselves." (FG2 acute)<br>"There is a case for having the walls padded permanently, we usually put, I was thinking of when we had the …, you put the mattresses against the wall. We've had that." (FG4 subacute)<br>"they've hit heads on the walls, they've hit their hands into the walls, all it, there's a case for it. … it's not unreasonable to have one room designated." (FG4 subacute)<br>"we're not talking about locking someone in those rooms or whatever…we're talking about protecting them while they're so agitated." (FG4 subacute)

**Structure, systems and processes**	Acute setting often has to focus on keeping patients alive	"with the acute setting they have to think of the acute, because that's what they think of… Whereas with us we've got the rehab to get them home or their destination." (FG3 subacute)<br>"The turnover is incredible … patterns of nursing … five minutely off/hourly off, this room, that room, this room, that room – it's conveyor belt sort of." (FG3 subacute)<br>"I think we have the capacity to do those more proactive approaches, whereas the acute's very much reactive." (FG3 subacute)
**Structure, systems and processes**	Clustering nursing care can minimise triggering behaviours	"minimising the nursing interventions sometimes. If the patient is sleeping and they're fine, they're fine. Just leave them alone.… Cluster care." (FG1 acute)<br>"They're fast asleep when the very thing they need is sleep not to do their obs…The doctors have gone home and then you're left wondering do they want strict four-hourly or is it okay if I'm flexible with the patient." (FG1 acute)
**Structures, systems and processes**	Consistent nursing can be key to identifying behaviour triggers and strategies	"nursing staff that can be key nursing staff for those people with difficult behaviours and provide some consistency for those people so they can get to know those people, build that rapport, figure out what some of the triggers are with that team." (FG3 subacute)<br>"We don't let the casuals do our prominent one to ones, very behavioural because when you've got the one to ones and it's the regular team they know how to carry on with strategies and things like that" (FG3 subacute)

		"And then they [patient] just sometimes might get that rapport with certain staff which is not always fair for them to work with that patient all the time, but it does help behavioural wise sometimes." (FG2 acute)
**Structures, systems and processes**<br>**Resources (recipients)**	Continuity of staffing can be difficult	"putting the same staff in with the same patient doesn't happen very often because of our, just staff that work part-time and different hours and things like that. So those patients don't get that continuity and get sort of that familiarity with those staff members as well." (FG2 acute)<br>"continuation of care as well, sometimes it just goes the other way because staff will say, I can't do, I can't do another shift looking after that patient. I can't, I've done 2 shifts and it's just not going." (FG2 acute)
**Structure, systems, processes**	Effective handover of what works well	"find what works best and then communicate that to your other, to the other staff." (FG2 acute)<br>"you really need to get a good handover about all of that otherwise you're kind of just starting from scratch, trying to work out" (FG2 acute)
**Structure, systems and processes**	A structured timetable for scheduled therapy and rest breaks	"It is good to have some structure and then it highlights the rest, so often we give the education that it is important, that rest is part of the recovery. So it's important to give time for rest and that protected time when they don't have visitors, otherwise it's just constant." (FG2 acute)<br>"we do use these timetables when we have the right patient and we do schedule it in there, like rest and block different therapy…so then

		they can see a bit of structure and that can help visitors plan good times to come as well." (FG acute)<br>"when we have the patients that are here for a while and they're in that stage where they're having quite a few therapies we do prioritise trying to keep to that time" (FG2 acute)
**Structure, systems and processes**	Code blacks can be a proactive approach for help	"Code black sometimes means I need a hand. I need an extra hand or I need to step away and some change of face situation." (FG3 subacute)<br>"I think there's this idea that we aren't doing a good job because we still have code blacks." (FG3 subacute)
**Staffing and funding**	Reducing restraints and medications requires more funding for staffing resources	"if you want us to use less medications, less restraints all the rest of it, we need the funding to have the increased staffing that needs to come to use those strategies." (FG3 subacute)<br>"psychology/neuropsychiatry in acute and sub-acute settings that is what you're going to replace, those restraints with extra staff. And to be honest, occupational therapy … as well, but those two are the essentials." (FG3 subacute)
**Staffing and funding**	Neuropsychiatry services are needed in the acute hospital	"It has to be really bad to get neuropsychiatry on the ward." (FG1 acute)<br>"It gets so bad by the time they [neuropsychiatry] actually come here." (FG1 acute)<br>"And that could be because [this hospital] doesn't have their own neuropsychiatrist…We have to get it from [another hospital] and it has to have – we have to get funding and it has to be

		approved…Seems a bit strange, doesn't it? Like you said, it's a major … hospital" (FG1 acute)<br>"if we want it [neuropsychiatry] we can ask and I think sometimes source it privately but it's difficult to get to hold of and sometimes there's a wait." (FG2 acute)<br>"it seemed like we were able to get them [neuropsychiatrist] when the patients were at an extreme level of behaviour, so it wasn't like a preventative, it was when they were either really low conscious or really, really aggressive." (FG2 acute)<br>"It's just the lack isn't there, there's a lack of available neuro-psychology or neuro-psychiatry." (FG2 acute)<br>"it's actually quite hard to recruit to some of those positions [neuropsychiatrist] because they're actually quite hard to find that sort of experience and then keep them" (FG4 subacute)
**Staffing and funding**	Neuropsychiatry input results in more settled patients	"what we've found is the sooner neuropsychiatry come in and change different medications (propanalol, gabapentin) actually some of the agitation calms right down" (FG1 acute)<br>"We see a difference within 24 hours of them coming and seeing them." (FG1 acute)<br>"once they had neuropsych[iatry] then they seemed to settle down." (FG1 acute)<br>"for the time frames of how long it takes for them to come review and often we see a very quick change in one dose." (FG1 acute)

**Staffing and funding**	Neuropsychology services are not available for TBI patient in the acute hospital	"We did have neuro-psychology and she left… they never got another person in so I don't know. It's like a lack of those people." (FG1 acute)<br>"we used to have some neuropsychology but we don't have that now" (FG2 acute)
**Culture**<br>**Feeling valued**	Staff feel unsupported in their roles	"At the end of the day everyone knows nobody's going to look after you, you just have to look after yourself." (FG1 acute)<br>"We're in the caring profession but no-one cares for us." (FG1 acute)<br>"You don't feel supported by those above or valued and your skills." (FG1 acute)<br>"I mean we all work hard as a team but it's the above that don't really care." (FG1 acute)<br>"Just a verbal "Hey we appreciate what you're doing guys". (FG1 acute)<br>"Even just having someone acknowledge what we do, I know that sounds bizarre, that makes me feel better too." (FG4 subacute)
**Feeling valued**	Supportive leadership is important to staff	"You've got to have a strong team who understand this and know when to step in and step out; it does help. It helps when your manager or someone says how are you feeling, are you okay?" (FG3 subacute)<br>"Leadership that get it is so important." (FG3 subacute)

**Staffing and funding**<br>**Culture**	It is difficult to find staff to work on the ward	"Before COVID and stuff when we used to get agency and stuff like that, they would refuse to come to [this ward]… Because being this ward is like spinal patients and it's really heavy and then they see these behaviours" (FG1 acute)<br>"People are doing doubles and then they're pulled off the next shift so then you're running behind, one short in the morning or whatever." (FG2 acute)<br>"staffing issue in general have been tough. Nearly twice a week we're one down" (FG1 acute)<br>"We recently had a staff member leave because of a TBI [patient], the way it must've triggered something in her and she never came back again." (FG2 acute)
**Staffing and funding**	1:1 special comes from within staffing ratios	"If we have the one to one, which we often do, we do it within our numbers." (FG1 acute)<br>"Yeah we'll get the one on one but that means it's one less staff member for the rest of the ward." (FG1 acute)
**Staffing and funding**	Allocating experienced staff to TBI patients	"the person allocating to someone with particular behaviours, who have a lot of experience, and that's thought through quite a bit about who's going to be allocated to that person." (FG1 acute)<br>"We will very rarely put a reliever with a known aggressive patient." (FG1 acute)<br>"we always try to do most of our TBIs with a regular staff." (FG2 acute)

		"That's it, just the support from those around you I guess and knowing each other's strengths and weaknesses. Some people can interact with that client, some people can't. So allocations are kind of looked at that way" (FG3 subacute)
**Structure, systems, processes**	Delays in assistance during crisis point	"We've had a situation where the patient's being really-really aggressive so we've had to call a code black and then we've had a phone call saying they can't come at the moment, they're at other code blacks. What do we do?" (FG1 acute)<br>"it seemed like a few minutes was half a hour. It was just chaotic." (FG1 acute)<br>"we're exhausted, we're fatigued, there's no point in calling a code because no one turns up anymore because it was happening so often." (FG3 subacute)
**Structure, systems and processes**<br>**Staffing and funding**	Effective behaviour management is resources intensive	"To effectively manage behaviour is insanely resource intensive. Collecting the data we have barriers here. In acute I can only imagine ten fold of trying to document – you don't have time as a clinician, you've got to document already in one place and then you fill out a behaviour log and you need everybody to do that and it never gets done comprehensively and then you need someone to sit down and sift through that data which takes ridiculously long, you need to look for patterns, you need to analyse, you need to liaise with everybody about the same behaviour what works/what doesn't; it's unbelievably time intensive. We see the value of that absolutely and we have the

		resources and the design and the system setup that allows for that, but yeah you need to have a huddle with the whole treating team to bounce off each other and explore what works" (FG3 subacute)
**Policies and priorities**	Documentation is needed for use of restraints	"the protocols around that have changed quite a lot it has to be documented by a medical staff, there have to be – we document it every 24 hours with us as well." (FG2 acute)<br>"But then if you don't fill out the proper paperwork as well, we get into massive trouble" (FG1 acute)<br>"definitely have the review process where it's constantly reviewed and checked." (subacute)
**Policies and priorities**<br>**Research evidence - innovation**	Lack of clinical practice guidelines	"So what I've found from my practice and research and reading about this area, is that there's not a lot of …very specific international and Australian guidelines that you do this, you do this, you do this, you do this." (FG2 acute)
**Policies and priorities**	Strategic directions do not reflect the practice in the unit	"I get a bit frustrated I think that outside of our unit there's all this push to create behaviour education strategies blah, blah, blah and then some of that's coming back people looking at our numbers of code blacks and what not; I don't think people don't realise how much we do here and all of the processes that we have around behaviour, the resources we need and I think I get frustrated it's very disjointed within the network." (FG3 subacute)
