# Supplementary material for: Barriers and enablers to managing challenging behaviours after traumatic brain injury in the acute hospital setting: a qualitative study
Source: BMC Health Serv Res. 2023 Nov 16;23:1266. doi: 10.1186/s12913-023-10279-z (PMC10655469; doi:10.1186/s12913-023-10279-z)
Supplement: Supplementary file 3 — Supplementary Material 3 [file 12913_2023_10279_MOESM3_ESM.pdf]

Supplementary File 3: Constructs of the i-PARIHS implementation framework utilised for data collection, data analysis and mapped themes.

**Constructs of i-PARIHS framework**

**Innovation**

Evidence for TBI behaviour management

**Recipients**

Staff providing care and patients receiving care

**Context**

Ward level, organisational, and policy characteristics

**Focus group questions relating to constructs of i-PARIHS framework**

Focus group questions relating to:

- Current evidence and guideline recommendations
- Relevance of the evidence to the acute context

Focus group questions relating to:

- Staff skills and knowledge
- Confidence in decision making

Focus group questions relating to:

- Hospital environment and resources
- Leadership, feeling valued, teamwork, culture
- Workforce, strategic priorities

**Data analysis**

**Inductive-deductive coding and mapping to i-PARIHS constructs**

**Themes identified as barriers and enablers mapped to characteristics of i-PARIHS constructs**

Innovation Barrier:

Limited evidence to inform clinical decision making

Recipient Barrier:

Lack of experienced multidisciplinary staff with practical skills

Context Barrier:

Hospital systems and resources

Recipients and Context Enabler:

Consistent, person-centred care

Recipient Enabler:

Supportive teams

Harvey, G., Kitson, A. PARIHS revisited: from heuristic to integrated framework for the successful implementation of knowledge into practice. *Implementation Sci* **11**, 33 (2015).
https://doi.org/10.1186/s13012-016-0398-2
